# Multiregional Radiomic Signatures Based on Functional Parametric Maps from DCE-MRI for Preoperative Identification of Estrogen Receptor and Progesterone Receptor Status in Breast Cancer

**DOI:** 10.3390/diagnostics12102558

**Published:** 2022-10-21

**Authors:** Shiling Zhong, Fan Wang, Zhiying Wang, Minghui Zhou, Chunli Li, Jiandong Yin

**Affiliations:** Department of Radiology, Shengjing Hospital of China Medical University, Shenyang 110004, China

**Keywords:** radiomics, estrogen receptor, progesterone receptor, breast cancer, magnetic resonance imaging

## Abstract

Radiomics based on dynamic contrast-enhanced magnetic resonance imaging (DCE-MRI) has been used for breast estrogen receptor (ER) and progesterone receptor (PR) status evaluation. However, the radiomic features of peritumoral regions were not thoroughly analyzed. This study aimed to establish and validate the multiregional radiomic signatures (RSs) for the preoperative identification of the ER and PR status in breast cancer. A total of 443 patients with breast cancer were divided into training (*n* = 356) and validation (*n* = 87) sets. Radiomic features were extracted from intra- and peritumoral regions on six functional parametric maps from DCE-MRI. A two-sample *t*-test, least absolute shrinkage and selection operator regression, and stepwise were used for feature selections. Three RSs for predicting the ER and PR status were constructed using a logistic regression model based on selected intratumoral, peritumoral, and combined intra- and peritumoral radiomic features. The area under the receiver operator characteristic curve (AUC) was used to assess the discriminative performance of three RSs. The AUCs of intra- and peritumoral RSs for identifying the ER status were 0.828/0.791 and 0.755/0.733 in the training and validation sets, respectively. For predicting the PR status, intra- and peritumoral RSs resulted in AUCs of 0.816/0.749 and 0.806/0.708 in the training and validation sets, respectively. Multiregional RSs achieved the best AUCs among three RSs for evaluating the ER (0.851 and 0.833) and PR (0.848 and 0.763) status. In conclusion, multiregional RSs based on functional parametric maps from DCE-MRI showed promising results for preoperatively evaluating the ER and PR status in breast cancer patients. Further studies using a larger cohort from multiple centers are necessary to confirm the reliability of the established models before clinical application.

## 1. Introduction

Female breast cancer (BC) has become the most commonly diagnosed cancer, with an estimated 2.3 million new cases in 2020 [1]. BC is a hormone-dependent disease [2]. The expressions of estrogen receptors (ERs) and/or progesterone receptors (PRs) are present in approximately 70%–80% of BCs, and are associated with other tumor characteristics [2,3,4]. In addition, the ER and PR status are important markers for determining luminal subtypes, treatment choice, and the prognosis [5,6]. In clinical practice, gene status is evaluated using immunohistochemical analyses of tissue samples obtained from core needle biopsies, which are invasive and time-consuming procedures [7]. However, the time of the first diagnosis and treatment is of crucial importance for patients with BC [8]. A study conducted by Sitaula et al. proposed a new pathological diagnosis method, combining foreground and background features at histopathological image parts and whole levels extracted from two mid-level pooling layers of pre-trained VGG16 models, which yields a better effectiveness and efficiency for BC classification [9]. This technique might be also suitable for the evaluation of the ER and PR status in BC patients using histopathology images. However, that method still requires a needle biopsy to obtain histopathology images. Therefore, there is an urgent need to develop a noninvasive and preoperative model to identify the ER and PR status.

Radiomics is a promising new methodology, which can characterize tumor heterogeneity by extracting a large number of quantitative features from medical images [10,11]. Previous studies have shown that radiomic features derived from contrast-enhanced spectral mammography, or magnetic resonance imaging (MRI) data have the potential to identify the ER and PR status of BC [8,12,13]. However, these radiomic studies mainly focused on intratumoral regions and ignored the surrounding tissues. Some evidence has suggested that tumors are not only comprised of tumor cells, but also host stromal cells, which may provide additional valuable information to diagnose BC [14,15,16,17]. In addition, two recent studies on radiomics found that the models established using radiomic features derived from intra- and peritumoral region in functional parametric maps, which are calculated by dynamic contrast-enhanced MRI, achieved a better diagnostic ability than the models based on intratumoral features for the prediction of Ki-67, HER-2, and sentinel lymph node metastasis [18,19]. Therefore, functional parametric maps can perform semi-quantitative analysis for intra- and peritumoral regions and capture the change existed in these regions. Moreover, new evidence showed that the expression of estrogen and progesterone signals in the tumor microenvironment was also different [20]. However, to the best of our knowledge, to evaluate the ER and PR status in BC patients, there has been no radiomic study based on multiregional features from functional parametric maps. Thus, a hypothesis that combines intra- and peritumoral features might yield a better performance for evaluating hormonal status is proposed.

In this study, we aimed to establish and validate the multiregional radiomic signatures (RSs) based on functional parametric maps from DCE-MRI for the preoperative identification of the ER and PR status of BC patients.

## 2. Materials and Methods

### 2.1. Patients

The Institutional Ethics Committee approved this retrospective study, and the requirement for informed consent was waived. A total of 443 female patients with pathologically confirmed BC were selected for this study from November 2017 to February 2021. The inclusion and exclusion criteria of the patients are provided in the Supplemental Material. The recruitment pathway for the selected patients is shown in Supplemental Figure S1.

**Figure 1:**
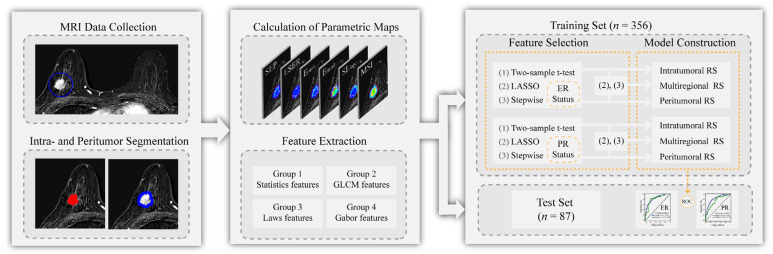
Flowchart of approaches used in this study.

All patients were assigned into two cohorts at a ratio of 4:1 based on the time of treatment [21]. The training set contained 356 patients who received treatment between November 2017 and May 2020. The validation set contained 87 patients who received treatment between June 2020 and February 2021. Figure 1 provides the flowchart of approaches used in this study.

### 2.2. Pathological Evaluation

The gene status of each patient was evaluated by pathologists using immunohistochemical analyses. The ER or PR status were recognized as positive if at least 1% positive tumor nuclei were present in the sample, or otherwise deemed negative [22]. A Ki-67 expression level ≥14% was defined as high, or otherwise was defined as low [23].

### 2.3. MRI Acquisition

A Signa HDxt 3.0 T MRI system (GE Healthcare Life Sciences, Chicago, IL, USA) was used to perform the breast examinations of all patients with the prone position. A dedicated eight-channel double-breast coil was used for the DCE-MRI scans of all patients, which were transverse. For each scan, a pre-contrast series was initially performed and eight post-contrast series were then conducted after an intravenous injection of the contrast agent [0.5 mmol/mL of Omniscan™ (gadodiamide); GE Healthcare, Berlin, Germany] at 4 mL/s (0.15 mmol/kg body weight) with an equal volume of saline. The parameters of the scan were as follows: repetition time = 7.42 ms, echo time = 4.25 ms, flip angle = 15°, slice thickness = 2.20 mm, spacing between slices = 2.20 mm, inversion time = 20 ms, image matrix = 1024 × 1024, time per volume = 80 s, slice number = 78, and field of view = 340 × 340 mm^2^. Eight post-contrast images subtracted the pre-contrast images to obtain the images of eight subtraction.

### 2.4. Tumor Segmentation

The representative slice image with the maximum diameter of the tumor in the subtraction image was selected by two radiologists (Reader 1, with 10 years of diagnostic experience in breast cancer, and Reader 2, with 5 years of experience). Another senior radiologist (Reader 3 with 14 years of experience) performed the evaluation for cases when there was a disagreement between Readers 1 and 2. All readers were blinded to the clinical and histopathological data.

The intratumoral region of interest (ROI) was obtained using a semi-automatic segmentation method based on the maximum between-cluster variance by Reader 1. The detailed descriptions for the semi-automatic segmentation method are summarized in the Supplemental Material. The peritumoral ROI was acquired by dilating a radial distance of 4 mm from the boundary of the intratumoral ROI [19]. The procedure of tumor segmentation was performed based on MATLAB 2018a (Mathworks, Natick, MA, USA). In addition, for tumors near the edge of the breast or chest wall, a breast parenchyma ROI (mask) was manually created using ITK-SNAP software (www.itksnap.org) and loaded into MATLAB 2018a. The dilation of the peritumoral ROI was additionally limited within the boundary of the breast tissue so that it did not exceed the breast region [24].

### 2.5. Calculation of Functional Parametric Maps

For the intra- and peritumoral ROIs, six functional parametric maps, the maximum slope of increase (MSI), slope of signal intensity (SI_slope_), initial percentage of peak enhancement (E_initial_), early signal enhancement ratio (ESER), percentage of peak enhancement (E_peak_), and second enhancement percentage (SEP) maps, were calculated on a pixel-by-pixel basis according to the following equations.

MSI = max (SI_i+1_ − SI_i_) (1)

SI_slope_ = [(SI_8_ − SI_mean_)/SI_mean_] × 100% (2)

E_initial_ = (SI_1_ − SI_0_)/SI_0_ × 100% (3)

ESER = (SI_1_ − SI_0_)/(SI_2_ − SI_0_) × 100% (4)

E_peak_ = (SI_peak_ − SI_0_)/SI_0_ × 100% (5)

SEP = (SI_2_ − SI_0_)/SI_0_ × 100% (6)

where SI is the signal intensity of each pixel in the image, SI_0_ represents the value of the pixel in the pre-contrast image, SI (i), i = (1, 2, 3, 4, 5, 6, 7, 8), represents the value of the pixel in the i-th post-contrast scan, SI_mean_ is the mean value of the first two post-contrast time points, and SI_peak_ represents the image pixel value at the peak enhancement time point identified from the time-intensity curve.

### 2.6. Feature Extraction

The normalization between μ ± 3σ [μ: mean of image intensity within the ROI; σ: standard deviation (SD)] was performed for all pixel intensities of the intra- and peritumoral ROIs, and eight bits/pixel were used for the gray level range to change the signal-to-noise ratio of the texture results, prior to feature extraction [25,26,27]. Intra- and peritumoral ROIs of six functional parametric maps were used to calculate four categories of radiomic features including first-order statistic features, gray level co-occurrence matrix (GLCM) features, Laws features, and Gabor features. For each case, a total of 4980 radiomic features were extracted. Supplemental Table S1 provides the information of these features in detail. To eliminate the limitations, which were imposed by the units among the different feature, all features were normalized according to the corresponding formula [z distribution = (value – mean value)/SD)]. 

MATLAB 2018a was used to perform the image intensity normalization and feature extraction.

### 2.7. Interobserver Variability Assessment

Reader 1 and Reader 2 performed the ROI segmentation for 100 images, which were randomly selected. Radiomic features were then calculated for intra- and peritumoral ROIs of the segmented images of each radiologist. To assess the reproducibility and stability of the feature extractions, intraclass correlation coefficient (ICC) analysis was performed. Features with an ICC > 0.8 were deemed as stable features and chosen for subsequent radiomic analysis [28].

### 2.8. Feature Selection and RS Establishment

A three-step feature selection was used according to the ER and PR status for the intra- and peritumoral features of the training cohort, respectively. First, the features with *p* < 0.1 were primarily identified using the two-sample *t*-test [29]. Then, the least absolute shrinkage and selection operator regression (LASSO) based on 10-fold cross-validation was conducted [21]. Finally, a backward stepwise selection was performed, and the stopping rule was set to the likelihood ratio test with Akaike’s information criterion [30]. 

The optimal feature subsets for predicting the ER and PR status were selected for each region. The intratumoral radiomic score (intra-rad-score) and peritumoral rad-score (peri-rad-score) for identifying the ER and PR status were, respectively, calculated based on the selected features with nonzero coefficients, which were obtained using the logistic regression model. To establish the multiregional RSs for the ER and PR status, the selected intra- and peritumoral features were combined and once more fed into the LASSO and stepwise methods. Then, the multiregional radiomics scores (multi-rad-scores) for evaluating the ER and PR status were calculated using the logistic regression model based on the selected multiregional features.

The feature selection and RS establishment were performed by using R software (version 3.6.2).

### 2.9. Statistical Analysis

The area under the receiver-operator characteristic (ROC) curve (AUC) were used to evaluate the discriminative performance of three RSs for identifying the ER and PR status in both the training and validation sets. MedCalc software (version 14.10.20) was used to generate the ROC plots. The figure plots were performed using R software.

## 3. Results 

### 3.1. Patient Characteristics

**Figure 2:**
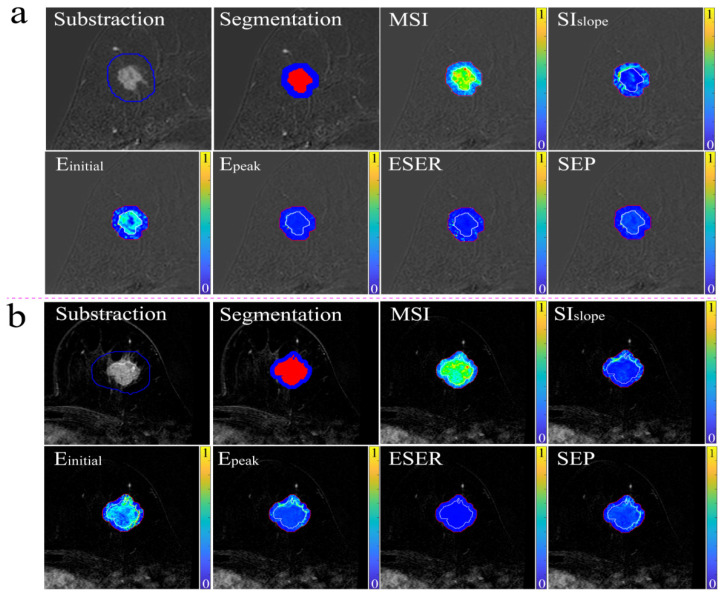
The lesion segmentation images and functional parametric maps (the intra- and peritumoral margins were marked using white and red lines) of two randomly selected cases. (**a**) The images of positive ER and PR status in BC. (**b**) The images of negative ER and PR status in BC.

A total of 443 BC patients (mean age: 51 years; age range: 23–85 years) were included in our study. The patient characteristics are shown in Table 1. Two randomly selected cases are shown in Figure 2, displaying the results of lesion segmentation along with six functional parametric maps.

### 3.2. Feature Selection and RS Establishment

In all, 1719 intratumoral and 1712 peritumoral features were shown to have a good stability. After feature selection with a two-sample *t*-test, LASSO and stepwise, 13 intratumoral and seven peritumoral features were selected to calculate the intra- and peri-rad-scores for identifying the ER status. To calculate the multi-rad-score for predicting the ER status, 17 multiregional features were chosen. Then, the three rad-scores were calculated using the corresponding coefficients of the logistic regression model. Detailed information of those selected features for identifying the ER status is provided in Supplemental Table S2. The distributions of the three rad-scores and ER status in the training and validation sets are shown in Supplemental Figures S2–S4.

For predicting the PR status, 17 intratumoral and 13 peritumoral features were chosen to calculate the intra- and peri-rad-scores. Next, 20 multiregional features were selected for the calculation of the multi-rad-score. Thereafter, the three rad-scores were calculated based on the corresponding logistic regression coefficients of the selected features. Detailed information of those selected features is summarized in Supplemental Table S3. The distributions of the three rad-scores and ER status in the training and validation sets are presented in Supplemental Figures S5–S7.

### 3.3. Radiomic Assessment

**Figure 3:**
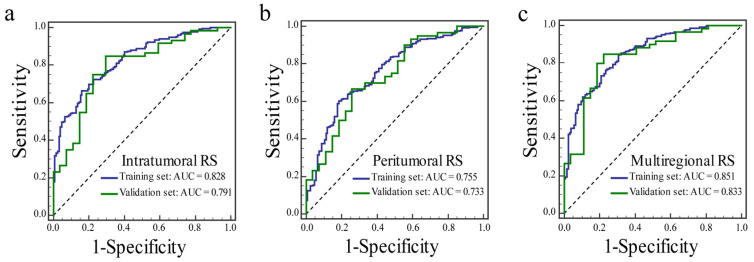
The receiver operator characteristic curves of the intratumoral RS (**a**), the peritumoral RS (**b**), and the multiregional RS (**c**) for predicting estrogen receptor status in the training and validation sets.

The ROC curves of the three RSs for identifying the ER status in the training and validation sets are shown in Figure 3. The diagnostic performances of three RSs are shown in Table 2. The multiregional RS achieved the best AUC among the three RSs in the training (AUC: 0.851) and validation (AUC: 0.833) sets. The intratumoral RS yielded an AUC of 0.828 in the training set and 0.791 in the validation set. The AUCs of the peritumoral RS were 0.755 and 0.733 in the training and validation sets, respectively.

**Figure 4:**
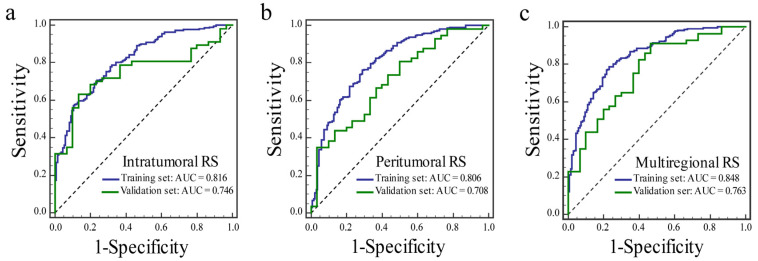
The receiver operating characteristic curves of the intratumoral RS (**a**), the peritumoral RS (**b**), and the multiregional RS (**c**) for predicting the progesterone receptor status in the training and validation sets.

For the prediction of the ER status, the ROC curves of three RSs in the training and validation sets are presented in Figure 4, and the diagnostic performances of the three RSs are provided in Table 3. The AUC of the multiregional RS was the best among the three RSs in the training (AUC: 0.848) and validation (AUC: 0.763) sets. The intratumoral RS obtained an AUC of 0.816 in the training set and 0.746 in the validation set. The peritumoral RS yielded an AUC of 0.806 in the training set and 0.708 in the validation set.

## 4. Discussion 

In the present study, the intratumoral and peritumoral RSs for identifying the ER and PR status were established using the intratumoral and peritumoral features extracted from six functional parametric maps of DCE-MRIs. Then, the multiregional RSs were constructed based on the combination of the selected intratumoral and peritumoral features for predicting the ER and PR status. The results showed that the multiregional RSs yielded the highest AUC values among the three RSs for evaluating the ER and PR status in both the training and validation sets.

ER and PR status are important clinical markers for the molecular subtyping, prognoses, and treatment of BC [5,6]. However, these markers are currently assessed through a diagnostic biopsy, which is an invasive procedure. Radiomics is an advanced technology which can transform medical images into high-dimensional and mineable data, and provide support for decision-making in oncology at a low cost and noninvasively through subsequent data analyses [31]. Some studies have reported the diagnostic performances of radiomics for the prediction of the ER and PR status in BC [12,13]. A study investigated the performance of the radiomic features from contrast-enhanced spectral mammography for identifying the ER and PR status of 68 breast lesions, which showed good diagnostic abilities [12]. However, MRI has been widely used in BC patients to define the extent of lesions, detect contralateral and occult diseases, and has been recommended for screening high-risk women [32,33]. A radiomic study by Monti et al. analyzed the DCE-MRI pharmacokinetic maps of 49 patients and established radiomic models, which showed promise for the discrimination of ER and PR status [13]. However, these radiomic studies included smaller samples and lacked an independent validation cohort. In addition, only radiomic features of the intratumoral regions were investigated in these studies. Two recent studies showed that the radiomic features of the peritumoral regions in functional parametric maps had a diagnostic value for the pathological findings of BC, such as Ki-67, HER-2, and sentinel lymph node metastasis [18,19]. In our study, the radiomic features were therefore extracted from the intra- and peritumoral regions in six functional parametric maps. To better evaluate and validate the models, our study included more samples compared with previous studies. Moreover, an independent validation cohort was divided and used to verify the effectiveness of the established models.

To improve the reproducibility of the radiomic models, features with a poor stability were excluded from our study. Then, a feature selection with two-sample *t*-tests, LASSO, and stepwise analyses was performed to select the optimal features from the stable features of each region, which were used to build the intratumoral and peritumoral RSs for a prediction of the ER and PR status, respectively. The results showed that the intra- and peritumoral RSs yielded a good diagnostic performance for the identification of the ER and PR status. To construct multiregional RSs, LASSO and stepwise methods were once again used. We found that the number of Gabor features was the largest among the features used to build the multiregional RSs, which are partially consistent with those of some previous studies [18,34,35,36]. This was mainly because Gabor features could detect the wavelike patterns of the intensity variations across different spatial scales in different orientations [35,36]. In addition, most of the selected features were derived from E_initial_ maps, which showed more useful details. Finally, we found that the multiregional RSs achieved higher AUC values compared with the intra- and peritumoral RSs in evaluating the ER and PR status. In addition, several recent studies also reported similar results that the radiomic models based on the multiregional features improved the diagnostic ability of pathological results for BC [18,19,34,35,36,37].

Computer-aided diagnosis (CAD) tools based on deep learning algorithms were successfully performed for the analysis of large and complex histopathological images, and achieved a good diagnostic performance and improved the work efficiency of pathologists [38,39]. A study by Sitaula et al. proposed a combined model for feature extraction to represent histopathology images capturing information in the images from different perspectives, and obtained a good accuracy in BC classification [9]. This method indicated that the pathological information of local lesions at the microscale is of vital importance for BC assessment. However, our study only investigated the diagnostic performance of the radiomic features of a macroscale tumor derived from functional parametric maps of DCE-MRI, ignoring this important information. In addition, one study demonstrated that radiopathomics, incorporating both radiological information of the whole tumor and pathological information of local lesions from a biopsy, could achieve a good performance in predicting discrepancies of the pathological response in rectal cancer [40]. Thus, in future research, we will analyze the features of a microscale tumor derived from BC histopathology images, and try to establish the radiopathomic models, combining the MRI features of a macroscale tumor and the histopathology features of a microscale tumor. 

This preliminary study had certain limitations. First, the sample size of the patient cohorts in our study was limited, and all cases were derived from a single center. To further validate the performance of our established models, more patients from multi-centers will be included in future research. Second, there may be a potential selection bias due to the retrospective design of this study. Third, only two-dimensional images of the largest tumor diameters were analyzed in our study. However, radiomics analyses based on the whole tumor volume may provide more comprehensive and important information about tumors, when compared with the models established using two-dimensional images. Fourth, some studies indicated that a radiomics model constructed using DCE-MRI combined with the apparent diffusion coefficient features could improve the diagnostic performance of the pathological results of BC [41,42]. However, the extraction of radiomic features in our study was only performed in the functional parametric maps based on the DCE-MRI, neglecting the features from apparent diffusion coefficient maps. Thus, multiple MRI sequences should be included in future radiomic studies. Finally, the pathological evaluation performed by multiple pathologists might exist inter-operator variability in the evaluation of IHC markers. In future research, we will carry out the relevant research to investigate the impact of inter-operator variability in the evaluation of IHC markers.

## 5. Conclusions

Our study developed multiregional RSs based on functional parametric maps from DCE-MRI, which showed favorable predictions of ER and PR status in BC patients. Further studies using a larger cohort from multiple centers are necessary to confirm the reliability of the established models before clinical application.

## Figures and Tables

**Table 1:** The characteristics of 443 BC patients.

Characteristics	Number of Patients (%)
Tumor size	
≤20	162 (36.57%)
>20	281 (63.43%)
ER status	
Positive	292 (65.91%)
Negative	151 (34.09%)
PR status	
Positive	279 (62.98%)
Negative	164 (37.02%)
Ki-67 level	
High	282 (63.66%)
Low	161 (36.34)
Histological type	
Invasive ductal carcinoma	394 (88.94%)
Invasive lobular carcinoma	15 (3.39%)
Ductal carcinoma in situ	17 (3.84%)
Phyllode carcinoma	11 (2.48%)
Papillary carcinoma	6 (1.35%)

**Table 2:** Diagnostic performances of three RSs for predicting the estrogen receptor status in the training and validation sets.

	Intratumoral RS		Peritumoral RS		Multiregional RS	
Metrics	Training Set	Validation Set	Training Set	Validation Set	Training Set	Validation Set
AUC (95% CI)	0.828 (0.784–0.865)	0.791 (0.691–0.871)	0.755 (0.707–0.799)	0.733 (0.628–0.822)	0.851 (0.809–0.886)	0.833 (0.738–0.905)
Sensitivity	0.664	0.850	0.603	0.667	0.845	0.850
Specificity	0.839	0.704	0.815	0.741	0.694	0.778

CI, confidence interval.

**Table 3:** Diagnostic performances of three RSs for predicting progesterone receptor status in the training and validation sets.

	Intratumoral RS		Peritumoral RS		Multiregional RS	
Metrics	Training Set	Validation Set	Training Set	Validation Set	Training Set	Validation Set
AUC (95% CI)	0.816 (0.772–0.855)	0.749 (0.645–0.836)	0.806 (0.761–0.846)	0.708 (0.600–0.800)	0.848 (0.806–0.883)	0.763 (0.660–0.848)
Sensitivity	0.707	0.632	0.761	0.351	0.788	0.912
Specificity	0.761	0.867	0.709	0.967	0.769	0.533

CI, confidence interval.

## Data Availability

The data supporting the results of this study can be obtained by contacting the corresponding authors. The data cannot be shared publicly due to privacy or ethical restrictions.

## Supplementary Materials

The following supporting information can be downloaded at: https://www.mdpi.com/article/10.3390/diagnostics12102558/s1, Figure S1: Recruitment pathway for patients selected in our study; Figure S2: Intra-rad-score for every patient in each cohort. (a) Intra-rad-score for every patient in the training set; (b) Intra-rad-score for every patient in the validation set. The status of ER was marked with different colors; Figure S3: Peri-rad-score for every patient in each cohort. (a) Peri-rad-score for every patient in the training set; (b) Peri-rad-score for every patient in the validation set. The status of ER was marked with different colors; Figure S4: Multi-rad-score for every patient in each cohort. (a) Multi-rad-score for every patient in the training set; (b) Multi-rad-score for every patient in the validation set. The status of ER was marked with different colors; Figure S5: Intra-rad-score for every patient in each cohort. (a) Intra-rad-score for every patient in the training set; (b) Intra-rad-score for every patient in the validation set. The status of PR was marked with different colors; Figure S6: Peri-rad-score for every patient in each cohort. (a) Peri-rad-score for every patient in the training set; (b) Peri-rad-score for every patient in the validation set. The status of PR was marked with different colors; Figure S7: Multi-rad-score for every patient in each cohort. (a) Multi-rad-score for every patient in the training set; (b) Multi-rad-score for every patient in the validation set. The status of PR was marked with different colors; Table S1: List of Radiomic Features; Table S2: Radiomic features identified in each region to discriminate ER positivity and negativity on functional parametric maps from breast DCE-MRI; Table S3: Radiomic features identified in each region to discriminate PR positivity and negativity on functional parametric maps from breast DCE-MRI.
