# Supplementary material for: Multiregional Radiomic Signatures Based on Functional Parametric Maps from DCE-MRI for Preoperative Identification of Estrogen Receptor and Progesterone Receptor Status in Breast Cancer
Source: Diagnostics (Basel). 2022 Oct 21;12(10):2558. doi: 10.3390/diagnostics12102558 (PMC9601361; doi:10.3390/diagnostics12102558)
Supplement: Supplementary file 1 [file diagnostics-12-02558-s001.zip › diagnostics-1933015-supplementary.pdf]

The inclusion criteria were (i) confirmed diagnosis of BC via pathological assessment of biopsy specimens; (ii) underwent DCE-MRI less than 1 month before surgery; and (iii) the presence of mass-like and single tumor (facilitating the subsequent segmentation of breast tumors).

The exclusion criteria were (i) preoperative treatment, such as radiotherapy, chemotherapy, or chemoradiotherapy; (ii) incomplete pathology data (ER or PR status unknown); (iii) lack of DCE-MRI data; and (iv) insufficient MRI quality (e.g., motion artifacts).

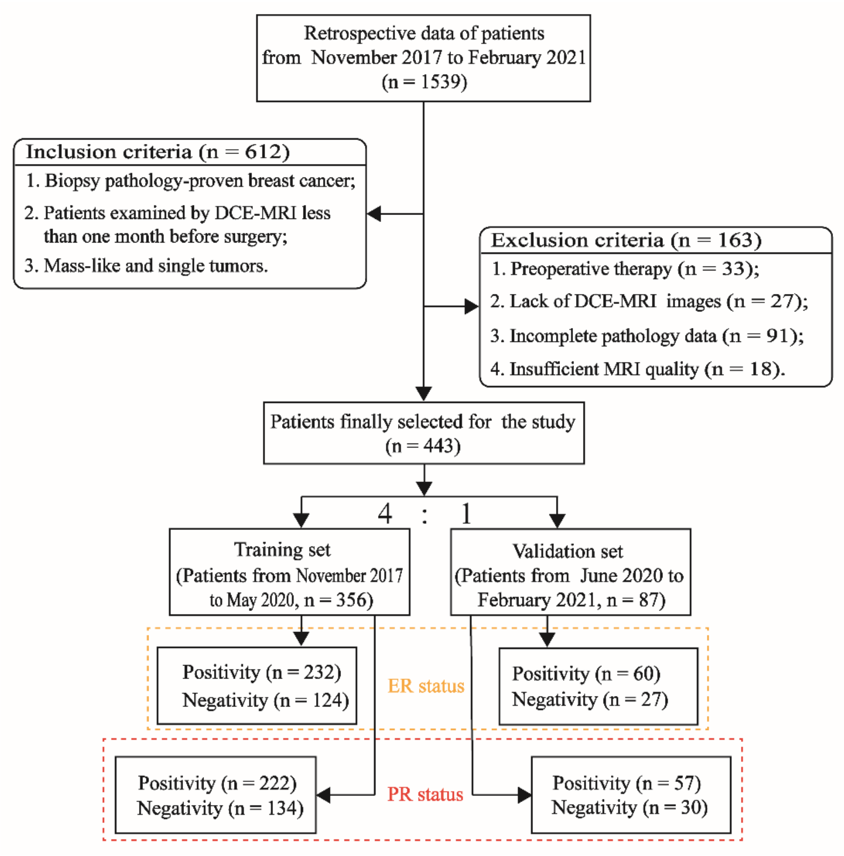

Figure S1. Recruitment pathway for patients selected in our study.

The semi-automatic segmentation procedures mainly involved the following steps. First, an arbitrary shaped ROI was drawn around the lesion area. Second, the maximum between-cluster variance method was applied to the ROI voxels and the segmented image was converted into a binary image with the objective region as 1 and the background region as 0. Third, morphological erosion was applied to the obtained binary image and the size of the structural element was set at 4 × 4. Fourth, a post-eroded image was traversed to obtain the largest unique eight-connected region. Finally, morphological dilation of the unique region was performed and the target region was considered as the intratumoral ROI.

Table S1. List of Radiomic Features

Category (quantity)	Radiomic features
First-order statistics n = 5	Mean, Median, SD, Skewness, Kurtosis.
GLCM n = 45[a]	Energy, Contrast, Correlation, Variance, Entropy, Homogeneity, Inverse difference moment, Information measures of correlation 1, Information measures of correlation 2.
Laws n = 125[a]	Response to 5-pixel × 5-pixel filter targeting the specific texture enhancement patterns in the X and Y directions. 25 descriptors derive from all combinations of five one - dimensional filters: level (L), edge (E), spot (S), wave (W), and ripple (R). L5 = [1 4 6 4 1], E5 = [-1 -2 0 2 1], S5 = [-1 0 2 0 -1], R5 = [1 -4 6 -4 1], and W5 = [-1 2 0 -2 -1]. The 25 filters were L5L5, L5E5, L5S5, L5W5, L5R5, E5L5, E5E5, E5S5, E5W5, E5R5, S5L5, S5E5, S5S5, S5W5, S5R5, W5L5, W5E5, W5S5, W5W5, W5R5, R5L5, R5E5, R5S5, R5W5, R5R5.
Gabor n = 240[a]	Gabor wavelet is sensitive to image edge and has good spatial locality and directional selectivity, and can grasp the spatial frequency (scale) and local structure characteristics of multiple directions in the local area of the image. Each descriptor quantifies response to a given Gabor filter at a specific frequency (f = 0, 2, 4, 8, 16, 32) and orientation (θ = 0°, 22.5°, 45°, 67.5°, 90°, 112.5°, 135°, 167.5°).

[a] First order statistics (mean, median, SD, skewness, and kurtosis) for per descriptor were calculated.
Abbreviations: GLCM, gray level co-occurrence matrix features

Table S2. Radiomic features identified in each region to discriminate ER positivity and negativity on functional parametric maps from breast DCE-MRI.

No.	Group	Descriptor	Statistic	Map	Location	Coefficient in LR model	
		Intratumoral radiomic features				Intra-rad-score	Multi-rad-score
I1	Gabor	f = 4, θ = 22.5°	Mean	MSI	Intratumor	- 0.7236	- 0.8257
I2	Gabor	f = 4, θ = 135°	Skewness	MSI	Intratumor	- 0.3938	- 0.4202
I3	Gabor	f = 64, θ = 135°	Skewness	MSI	Intratumor	- 0.3158	- 0.2919
I4	GLCM	Homogeneity	Mean	SI_{slope}	Intratumor	4.4488	4.5558
I5	GLCM	Inverse difference moment	Median	$E_{initial}$	Intratumor	0.2883	0.3851
I6	Gabor	f = 4, θ = 0°	Kurtosis	$E_{initial}$	Intratumor	- 0.2644	\
I7	Gabor	f = 16, θ = 22.5°	Median	$E_{initial}$	Intratumor	- 0.3855	- 0.3940
I8	Gabor	f = 4, θ = 112.5°	Median	$E_{initial}$	Intratumor	- 0.4357	- 0.3056
I9	Laws	L5E5	Median	$E_{initial}$	Intratumor	0.5190	0.3517
I10	Laws	E5L5	Skewness	$E_{initial}$	Intratumor	0.3956	0.4911
I11	Laws	W5S5	Median	$E_{initial}$	Intratumor	0.3361	0.3832
I12	GLCM	Homogeneity	Mean	ESER	Intratumor	0.9334	1.3239
I13	Gabor	f = 16, θ = 0°	Median	SEP	Intratumor	0.7591	0.7746
		Peritumoral radiomic features				Peri-rad-score	
P1	Gabor	G8	Skewness	MSI	Peritumor	- 0.2858	-0.3468
P2	GLCM	Variance	Median	SIslope	Peritumor	- 46.1874	- 83.0095
P3	GLCM	Information measures of correlation 2	Mean	SIslope	Peritumor	- 0.3372	0.6554
P4	GLCM	Correlation	Kurtosis	Einitial	Peritumor	0.2310	\
P5	Laws	L5E5	Skewness	Einitial	Peritumor	- 0.3938	- 0.3029
P6	Gabor	G64	Mean	ESER	Peritumor	- 0.2953	\
P7	Laws	W5R5	Kurtosis	ESER	Peritumor	- 0.21430	- 0.3678

Abbreviations: GLCM, gray level co-occurrence matrix features; SD, standard deviation; LR, logistic regression.

Intra-rad-score, peri-rad-score, and multi-rad-score calculation formulas in identifying ER status:

Intra-rad-score = 1.3084 - 0.7236 × I1 - 0.3938 × I2 - 0.3158 × I3 + 4.4488 × I4 + 0.2883 × I5 - 0.2644 × I6 - 0.3855 × I7 - 0.4357 × I8 + 0.5190 × I9 + 0.3956 × I10 + 0.3361 × I11 + 0.9334 × I12 + 0.7591 × I13

Peri-rad-score = - 1.3874 - 0.2858 × P1 - 46.1874 × P2 - 0.3372 × P3 + 0.231 × P4 - 0.3938 × P5 - 0.2953 × P6 - 0.2143 × P7

Multi-rad-score = - 2.5571 - 0.8257 × I1 - 0.4202 × I2 - 0.2919 × I3 + 4.5558 × I4 + 0.3851 × I5 - 0.394 × I7 - 0.3056 × I8 + 0.3517 × I9 + 0.4911 × I10 + 0.3832 × I11 + 1.3239 × I12 + 0.7746 × I13 - 0.3468 × P1 - 83.0095 × P2 + 0.6554 × P3 - 0.3029 × P5 - 0.3678 × P7

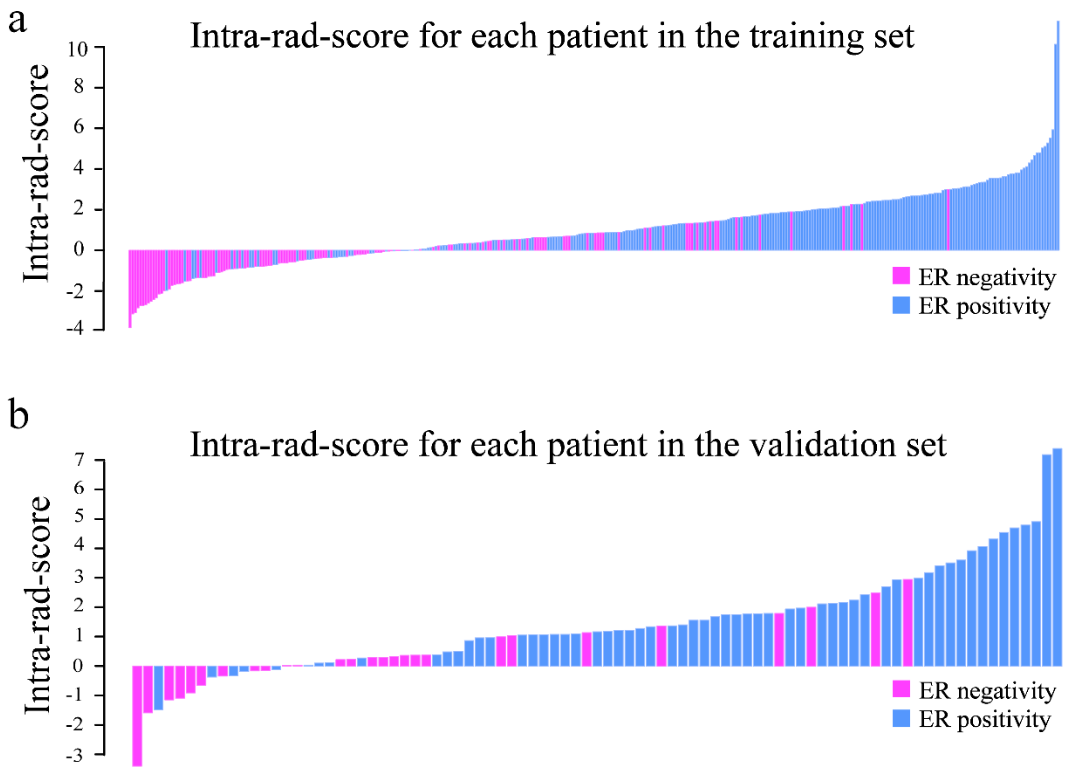

Figure S2. Intra-rad-score for every patient in each cohort. (a) Intra-rad-score for every patient in the training set; (b) Intra-rad-score for every patient in the validation set. The status of ER was marked with different colors.

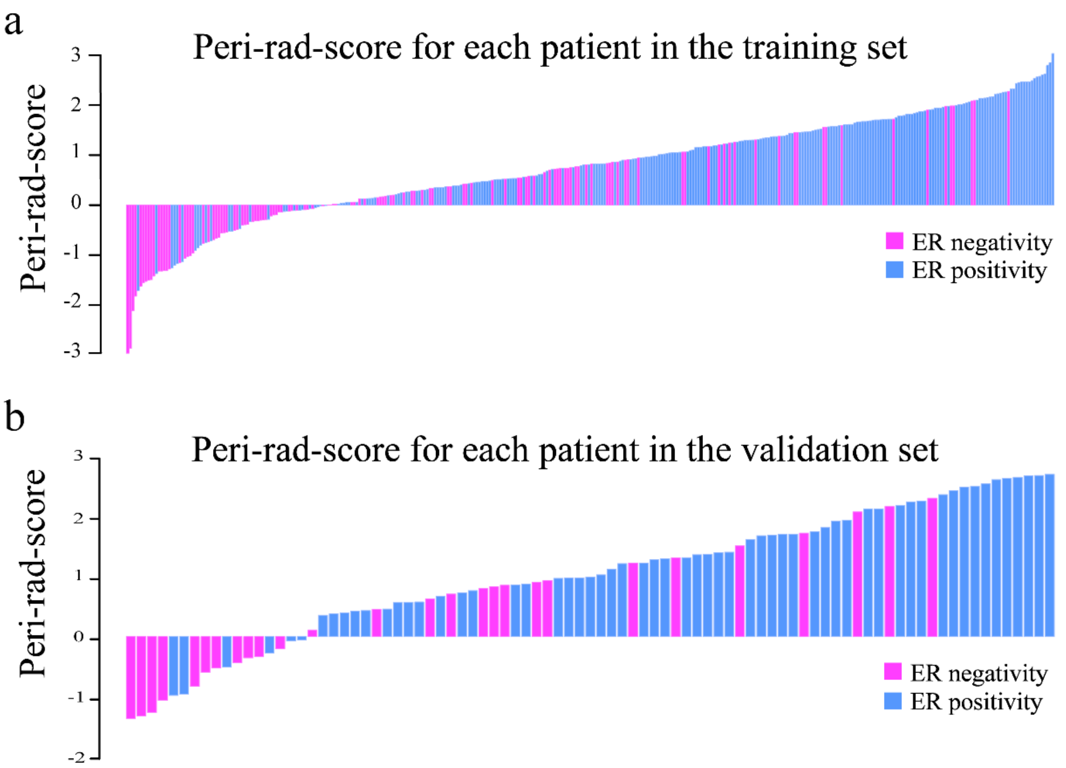

Figure S3. Peri-rad-score for every patient in each cohort. (a) Peri-rad-score for every patient in the training set; (b) Peri-rad-score for every patient in the validation set. The status of ER was marked with different colors.

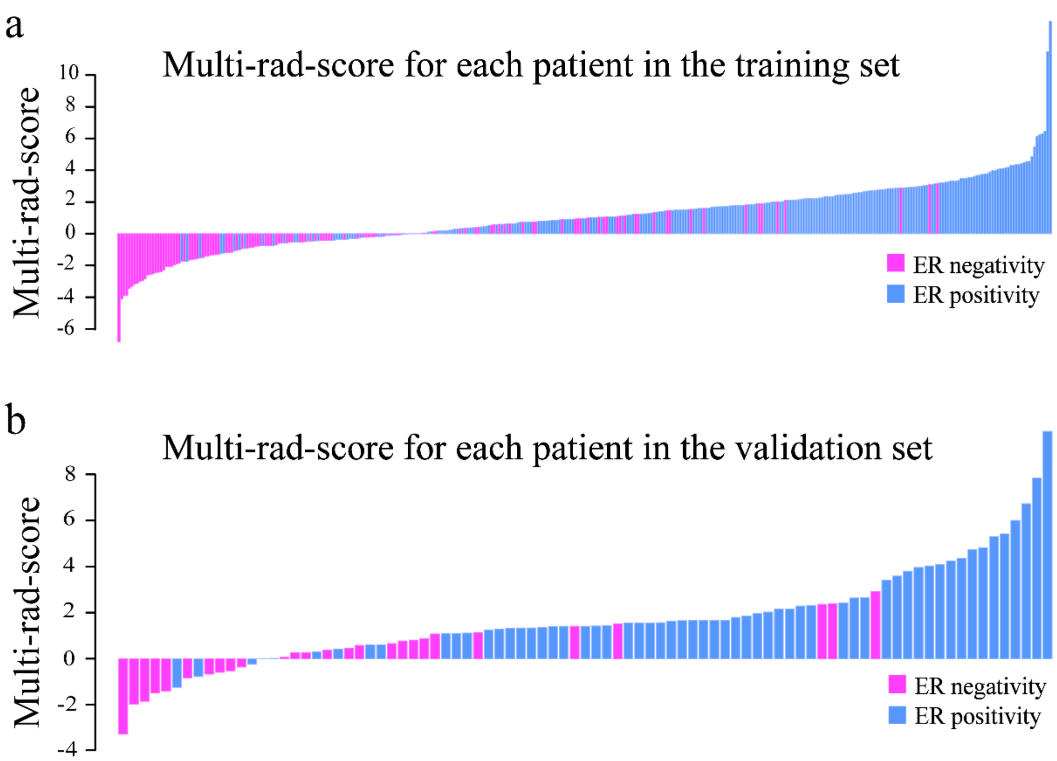

Figure S4. Multi-rad-score for every patient in each cohort. (a) Multi-rad-score for every patient in the training set; (b) Multi-rad-score for every patient in the validation set. The status of ER was marked with different colors.

Table S3. Radiomic features identified in each region to discriminate PR positivity and negativity on functional parametric maps from breast DCE-MRI.

No.	Group	Descriptor	Statistic	Map	Location	Coefficient in LR model	
		Intratumoral radiomic features				Intra-rad-score	Multi-rad-score
I1	Gabor	$f = 8, \theta = 22.5°$	Skewness	MSI	Intratumor	- 0.3091	\
I2	Gabor	$f = 4, \theta = 135°$	Skewness	MSI	Intratumor	- 0.4321	- 0.3956
I3	Laws	L5E5	Skewness	MSI	Intratumor	- 0.2763	- 0.2232
I4	Laws	W5L5	Skewness	MSI	Intratumor	0.3164	0.2783
I5	GLCM	Variance	Kurtosis	SI_{slope}	Intratumor	- 0.5552	\
I6	GLCM	Homogeneity	SD	SI_{slope}	Intratumor	0.4882	0.7970
I7	Gabor	$f = 64, \theta = 90°$	Skewness	SI_{slope}	Intratumor	- 0.3118	\
I8	GLCM	Inverse difference moment	Median	$E_{initial}$	Intratumor	0.5633	0.5584
I9	Gabor	$f = 16, \theta = 22.5°$	Median	$E_{initial}$	Intratumor	- 0.2972	- 0.2780
I10	Gabor	$f = 32, \theta = 67.5°$	SD	$E_{initial}$	Intratumor	0.6487	0.5464
I11	Gabor	$f = 4, \theta = 112.5°$	Median	$E_{initial}$	Intratumor	- 0.3814	- 0.4273
I12	Laws	E5L5	Skewness	$E_{initial}$	Intratumor	0.3105	0.4331
I13	Gabor	$f = 32, \theta = 45°$	Mean	ESER	Intratumor	- 0.3289	- 0.4358
I14	Gabor	$f = 64, \theta = 90°$	Mean	ESER	Intratumor	- 0.5183	- 0.5831
I15	Gabor	$f = 64, \theta = 135°$	Skewness	ESER	Intratumor	- 0.4025	- 0.6090
I16	Gabor	$f = 16, \theta = 0°$	Median	SEP	Intratumor	0.4850	0.4453
I17	Gabor	$f = 32, \theta = 112.5°$	Median	SEP	Intratumor	0.3602	0.4752
		Peritumoral radiomic features				Peri-rad-score	
P1	GLCM	Entropy	Mean	MSI	Peritumor	- 0.4363	\
P2	Laws	S5E5	Skewness	MSI	Peritumor	- 0.2205	\
P3	GLCM	Contrast	Median	SI_{slope}	Peritumor	- 0.3280	\
P4	Laws	L5L5	Median	SI_{slope}	Peritumor	- 0.6271	- 0.7345
P5	GLCM	Original	Skewness	$E_{initial}$	Peritumor	0.4465	0.4750
P6	Gabor	$f = 32, \theta = 112.5°$	Kurtosis	$E_{initial}$	Peritumor	- 0.3651	- 0.5293
P7	Laws	L5E5	Skewness	$E_{initial}$	Peritumor	- 0.2608	\
P8	Gabor	$f = 8, \theta = 45°$	Kurtosis	E_{peak}	Peritumor	- 0.2771	- 0.3845
P9	Gabor	$f = 32, \theta = 67.5°$	Median	E_{peak}	Peritumor	0.5859	0.4265
P10	Laws	S5E5	Skewness	E_{peak}	Peritumor	0.3427	0.3142
P11	Gabor	$f = 32, \theta = 45°$	Median	ESER	Peritumor	- 0.5521	\
P12	Gabor	$f = 64, \theta = 45°$	Mean	ESER	Peritumor	- 0.4427	\
P13	Gabor	$f = 4, \theta = 112.5°$	Median	ESER	Peritumor	0.3804	\

Abbreviations: GLCM, gray level co-occurrence matrix features; SD, standard deviation; LR, logistic regression.

Intra-rad-score, peri-rad-score, and multi-rad-score calculation formulas in identifying PR status:

Intra-rad-score = 0.8409 - 0.3091 × I1 - 0.4321 × I2 - 0.2763 × I3 + 0.3164 × I4 - 0.5552 × I5 + 0.4882 × I6 - 0.3118 × I7 + 0.5633 × I8 - 0.2972 × I9 + 0.6487 × I10 - 0.3814 × I11 + 0.3105 × I12 - 0.3289 × I13 - 0.5183 × I14 - 0.4025 × I15 + 0.4850 × I16 + 0.3602 × I17

Peri-rad-score = 0.7584 - 0.4363 × P1 - 0.2205 × P2 - 0.328 × P3 - 0.6271 × P4 + 0.4465 × P5 - 0.3651 × P6 - 0.2608 × P7 - 0.2771 × P8 + 0.5859 × P9 + 0.3427 × P10 - 0.5521 × P11 - 0.4427 × P12 + 0.3804 × P13

Multi-rad-score = 0.9540 - 0.3956 × I2 - 0.2232 × I3 + 0.2783 × I4 + 0.7970 × I6 + 0.5584 × I8 - 0.278 × I9 + 0.5464 × I10 - 0.4273 × I11 + 0.4331 × I12 - 0.4358 × I13 - 0.5831 × I14 - 0.609 × I15 + 0.4453 × I16 + 0.4752 × I17 - 0.7345 × P4 + 0.4750 × P5 - 0.5293 × P6 - 0.3845 × P8 + 0.4265 × P9 + 0.3142 × P10

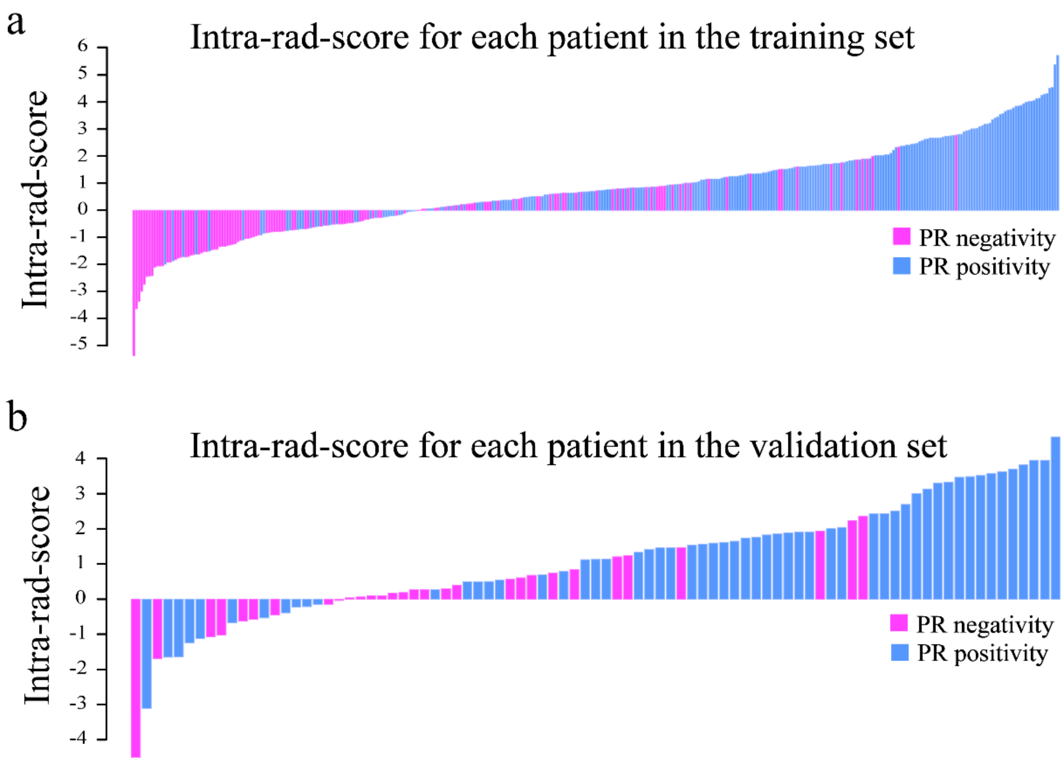

Figure S5. Intra-rad-score for every patient in each cohort. (a) Intra-rad-score for every patient in the training set; (b) Intra-rad-score for every patient in the validation set. The status of PR was marked with different colors.

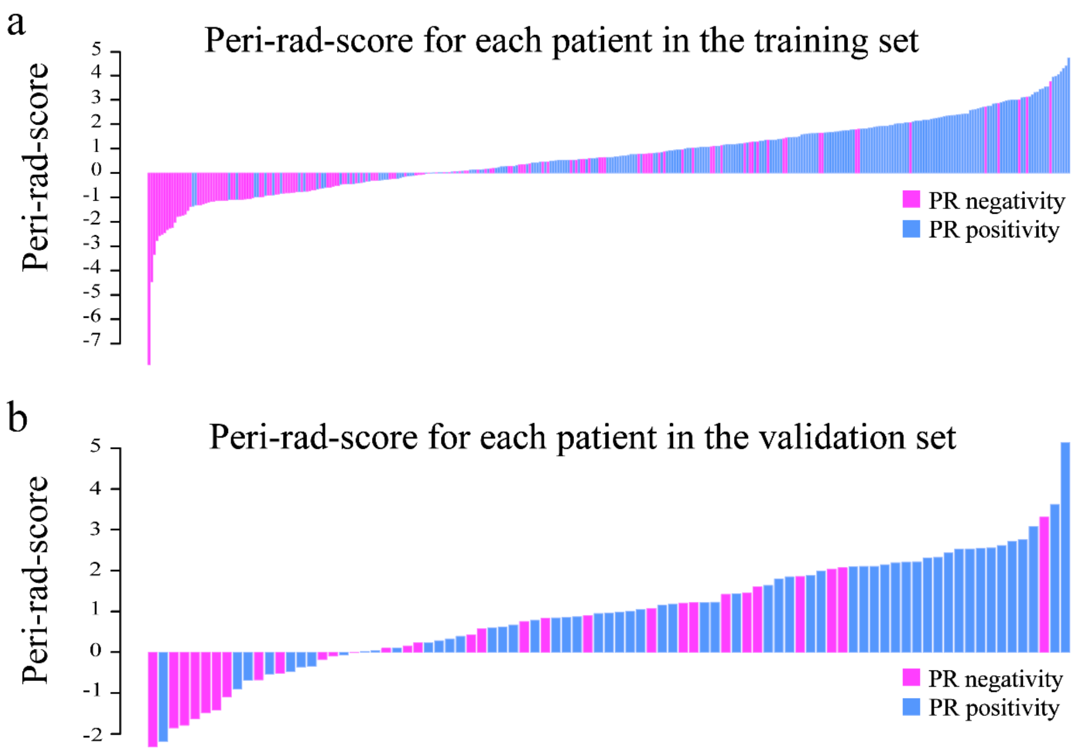

Figure S6. Peri-rad-score for every patient in each cohort. (a) Peri-rad-score for every patient in the training set; (b) Peri-rad-score for every patient in the validation set. The status of PR was marked with different colors.

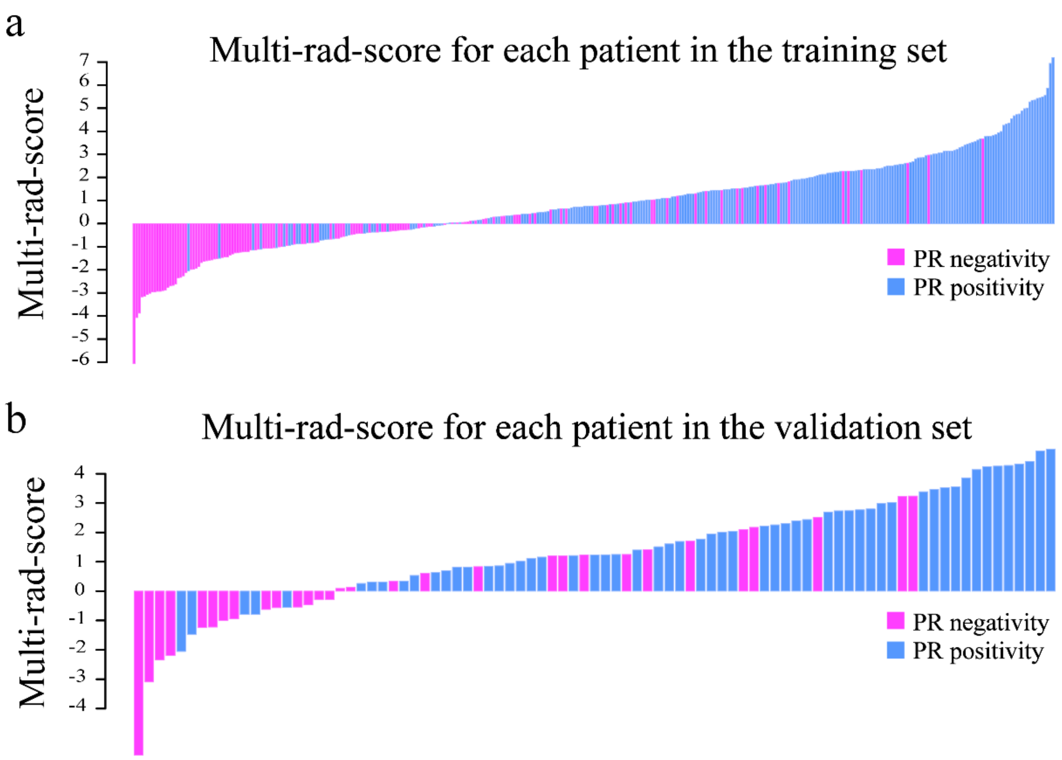

Figure S7. Multi-rad-score for every patient in each cohort. (a) Multi-rad-score for every patient in the training set; (b) Multi-rad-score for every patient in the validation set. The status of PR was marked with different colors.
